# Effect of *Oryctolagus cuniculus* (rabbit) rennet on the texture, rheology, and sensory properties of white cheese

**DOI:** 10.1002/fsn3.649

**Published:** 2018-04-17

**Authors:** Selin Alihanoğlu, Demet Ektiren, Çağım Akbulut Çakır, Hasan Vardin, Asliye Karaaslan, Mehmet Karaaslan

**Affiliations:** ^1^ Food Engineering Department Engineering Faculty Harran University Sanliurfa Turkey; ^2^ Technical Sciences Vocational School Food Technology Program Harran University Sanliurfa Turkey

**Keywords:** camel chymosin, cheese, rabbit rennet, rheology

## Abstract

Calf rennet has long been used in cheese‐making. Because of calf rennet shortage and high cost, novel proteases were needed to meet industry's increasing enzyme demand. Recombinant chymosins and camel chymosin were started to be used in the industry. There is no study in the literature subjecting use of rabbit rennet in cheese production. Chemical, rheological, and sensorial characteristics of white cheese made with rabbit rennet were investigated in this study. Quality characteristics of rabbit rennet cheese (RC) were compared to cheeses produced with commercial calf (CC) and camel chymosins (CLC). RC and CLC exhibited higher hardness and dynamic moduli values throughout the storage as compared to CC. Although moisture levels of cheese samples were similar at day 60, CC had much lower hardness and dynamic moduli values than CLC and RC. While the appearance and structure were better for CLC, the highest odor and taste scores were obtained by RC during 60 days of storage. The results of this investigation proposed that rabbit rennet could be a suitable milk coagulant for white cheese production. Our results showed that rabbit rennet has comparable cheese‐making performance with camel chymosin and could be a good alternative for calf chymosin.

## 1 INTRODUCTION

Rennet is a mixture of proteolytic–lipolytic enzymes (chymosin, pepsin, and lipase), and its activity is required for milk coagulation. Chymosin (EC 3.4.23.4) is key component of rennet and responsible for the degradation of casein proteins through disruption of covalent peptide bonds formed between Phe (105) and Met (106). Rennet is obtained from the abomasums of nursing animals. Many types of rennet are currently used in dairy industry including calf, pig, lamb, and goat, and recombinant chymosins are also available. In addition to animal sources, several proteases obtained from plants, for example, *Cynara cardunculus* (Verìssimo, Esteves, Faro, & Pires, 1995; Verìssimo et al., 1996), *Ficus carica*,* Arctium minus,* and *Solanum dobium,* are also employed in cheese‐making traditionally in some parts of the world (Robinson & Wilbey, 1998). Nursing calves had been considered as the primary rennet source in many regions of the world. However, calf‐originated rennet is not sufficient to supply dairy industry's coagulant demand. Therefore, investigation of novel coagulant sources has been underway (Houen, Madsen, Harlow, Lønblad, & Foltmann, 1996; Kappeler et al., 2006; Mohanty, Mukhopadhyay, Kaushık, Grover, & Batısh, 2003; Rogelj, Perko, Francky, Penca, & Pungerčar, 2001; Vega‐Hernández, Gómez‐Coello, Vıllar, & Claverıe‐Martín, 2004), and camel chymosin is already supplied to market for cheese production (Kappeler et al., 2006). There are studies in the literature reporting suitability of camel chymosin for the production of various cheese types (Bansal et al., 2009; Kappeler et al., 2006; Langholm et al., 2013; Moynihan et al., 2014; Soltani, Boran, & Hayaloglu, 2016).

Many natural proteinases have the potential to coagulate milk and form visible curd. However, the number of proteases that could be employed in cheese‐making is very limited. Chymosin, an aspartic proteinase, coagulates milk by specifically breaking the bonds established between amino acids present in the casein structure. Therefore, novel milk‐coagulating agents with a high specificity at the Phe(105)–Met(106) peptide bond and limited proteolytic activity are highly desirable by the industry. Good‐quality cheese production is related to high milk‐clotting activity and specificity of the enzyme on κ‐casein Phe(105)–Met(106) bond (Elagamy, 2000; Fox, 1969). Enzymes with low specificity may destroy other milk proteins and peptide bonds causing yield losses, formation of off‐flavors, excessive softening, and other functional defects. Excessive proteolysis during storage limits the shelf‐life of the cheese. For that reason, cheese industry is looking for enzymes with low proteolytic activity. Proteolysis results in formation of bitter peptides. These peptides are more dominant and tangible in reduced fat and salt cheeses, and use of chymosin with low proteolytic activity rather than the traditional calf rennet is required for those productions (Govindasamy‐Lucey, Lu, Jaeggi, Johnson, & Lucey, 2010).

Nowadays, scientists are still in pursuit of new coagulant sources for cheese production. Rabbit rennet has long traditionally been used in manufacturing of highly preferred cheeses in south‐east region of Anatolia. This type of cheese is demanded and consumed admiringly due to its superior taste and properties in storage period such as low bitterness and softening. It is suggested that rabbit abomasum could be a good source of rennet. However, this enzyme has not been investigated extensively for commercial exploitation (Rao & Dutta, 1981). This situation still maintains its continuity. The aim of this study was to evaluate the suitability of rabbit rennet in cheese production. For this purpose, three types of coagulants as rabbit rennet, calf chymosin, and camel chymosin were employed for the manufacture of white cheese. The cheese samples were assayed based on their chemical, rheological, and sensorial properties during 60 days of storage.

## 2 MATERIALS AND METHODS

### 2.1 Coagulants

The coagulants used in this research were calf chymosin (CHY‐MAX^TM^ M, 600 international milk‐clotting units (IMCU) per ml; Chr‐Hansen A/S, Hoersholm, Denmark), camel chymosin (CHY‐MAX^™^ M, 1000 IMCU/ml Chr‐Hansen A/S), and rabbit rennet extracted from young rabbit stomach. Rabbit stomach was obtained from local market, Mardin, Derik, which was cleaned, salted, and dried in a ventilated area prior to use in experiments and stored properly. Rabbit rennet extraction was carried out according to the method described by Lambert (1988). Briefly, 10 g of dried stomach tissue was soaked into 12% salt solution (1/10) (w/v) at room temperature and stirred on a magnetic stirrer for 5 min. After adjustment of pH of the mixture to 4.3 with 1 mol/L HCl, the solution was incubated at 35°C for 72 hr. The extract was filtered, and pH of the mixture was re‐adjusted to 5.6 with NaOH and stored at 4°C until cheese production.

### 2.2 Milk‐clotting activity determination

Milk‐clotting activities of enzymes were calculated according to Equation (1) to determine the amount of rabbit rennet, camel, and calf chymosin necessary to give a coagulum at desired cutting time. One milk‐clotting activity unit (MCA) was defined as the amount of enzyme required to clot 1 ml of substrate in 40 min at 35°C. The MCA was calculated using Equation (1):

(1) $$\text{Unit of milk}\text{‐}\text{clotting activity}\;\left(\text{MCA}\right)=\left(\frac{2,400}{t}\right)*\left(\frac{S}{E}\right)$$

where “*t*” is the time (s) necessary for clot formation, “*S*” is the milk volume, and “*E*” is the enzyme volume.

### 2.3 Cheese production

Three different types of coagulant were used in cheese production as rabbit rennet, commercial calf chymosin, and commercial camel chymosin. According to our preliminary tests, adding calf chymosin at the level of 600 IMCU/ml (0.6 ml diluted with 10 ml water) gave a coagulum with similar gel strength as camel chymosin at the level of 1,000 IMCU/ml (0.65 ml diluted with 10 ml water) and rabbit rennet extract (10.65 ml) at 60, 120, 120 min, respectively, for 45 L of milk. Cheese manufacturing carried out according to the method described by Hayaloglu, Guvena, and Fox (2002) with some modifications at Harran University Food Engineering Department laboratories. Prior to cheese production, milk (3% fat) was pasteurized at 63°C for 30 min., cooled down to 35°C temperature, pH was fixed to 5.9 using citric acid, and CaCl_2_ (0.02% w/v) was added. To obtain coagulum at desired cutting time (60 min at 35°C), rabbit rennet (10.65 ml), calf chymosin (0.6 ml), and camel chymosin (0.65 ml) were added to 45 L milk. After cutting the milk gel and draining the whey, curds were dry‐salted (2.25 g/L milk), pressed, and cold‐stored. Cheese samples were vacuum‐packed the next day and stored at 4°C for 60 days. Cheese‐making trial was performed in two replicates. Physicochemical, sensorial, rheological, and textural properties of the samples were evaluated at every 30 days.

### 2.4 Analysis of composition

The composition of cheese samples (pH, moisture%, protein%, fat%, salt%) was assessed on the day of production and at every 30 days during 60 days of storage period according to the method described by O'Mahony, Sousa, and McSweeney (2003). All analyses were performed on each cheese samples in triplicate.

### 2.5 Sensory analysis

Cheese samples were stored for 60 days at 4°C. Sensory characteristics of stored cheeses were evaluated by panelists at 0, 15, 30, 60 days of storage. A panel composed of 10 members evaluated the odor, taste, structure, inner and outer appearance of cheese with a 5‐point product‐specific descriptive intensity scale for each textural attribute. In this evaluation, 1 point represents “low” and 5 points represent “high” quality of cheese for each parameter examined. Flavor properties were defined according to Drake, McIngvale, Cadwallader, and Civille (2001) and Drake et al. (2005) and texture characteristics were evaluated according to Brown, Foegeding, Daubert, Drake, and Gompertz (2003).

### 2.6 Measurement of proteolysis

Proteolysis of the cheese samples was assessed by urea polyacrylamide gel electrophoresis (Urea‐PAGE) (12.5% total acrylamide, 4% cross‐linking agent, pH 8.9) according to the method of Andrews (1983). Urea‐PAGE gels were monitored using Bio‐imaging systems (mini BIS PRO, Israel) and photographed.

### 2.7 Measurement of the rheological and textural properties

The rheological properties of the cheeses were studied with a rheometer (Kinexus Pro+, Malvern Inst., Worcestershire, UK) using SAOS temperature sweep test as described by Govindasamy‐Lucey, Jaeggi, Johnson, Wang, and Lucey (2005) with small modifications. Cheese samples were cut into disks at 3 mm height and 20 mm diameter. Storage modulus (*G*′), loss modulus (*G*″), and loss tangent (LT) values were measured while heating the cheeses from 10 to 80°C at 1°C/30 s. A 20‐mm parallel plate was used, and the cheese was subjected to a strain of 0.5% at a frequency of 0.08 Hz. Texture analysis was performed using a Texture Analyzer TA‐XT2 (Stable Micro Systems, Godalming, Surrey, UK). Cheese samples were cut into 2 × 2 × 2 cm cubes. Texture profile analysis (TPA) was performed at 25% strain; texture parameters were calculated as previously described by Bourne (1978).

### 2.8 Statistical analysis

SPSS version 16 (SPSS Inc., Chicago, IL, USA) was used for analyzing the data obtained in this study. Analysis of variance (ANOVA) was employed to establish statistical differences between the physicochemical parameter values, texture profile analysis results, and sensory analysis scores according to rennet type, ripening time, and the interaction between those two factors.

## 3 RESULTS AND DISCUSSION

### 3.1 Cheese composition and yield

**Figure 1:**
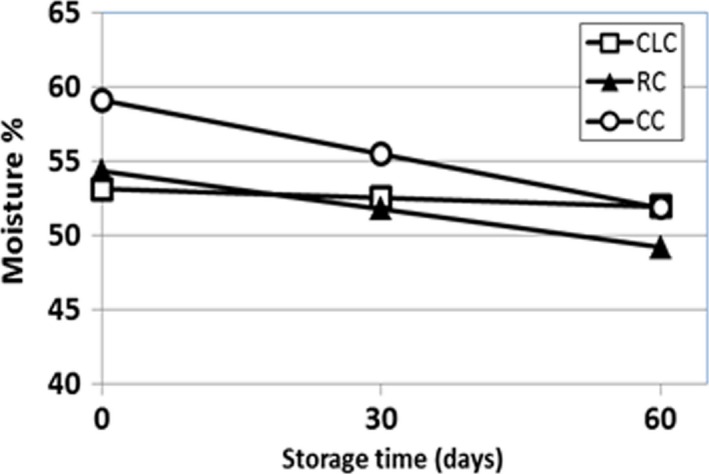
Moisture changes in white cheeses made with calf (CC), rabbit (RC), and camel coagulants (CLC) during 60 days of storage

Composition, yield, and pH values of the cheese samples are given in Table 1. No significant difference was observed at pH (5.8 ± 0.01) in rabbit rennet cheese (RC), commercial calf chymosin cheese (CC), and commercial camel chymosin cheese (CLC) samples at the day of production (*p* > .05). The pH values were gradually decreased during 60 days of storage. The similar pH values were observed in white cheese samples (Romeih, Michaelidou, Biliaderis, & Zerfiridis, 2002) during 90 day of storage. As no starter culture was used and no microbial growth was detected throughout the storage (microbiology results are not shown), decrease in pH was most likely caused by the change in ionic strength and buffering capacity with ongoing proteolysis and water release from cheese samples. Insoluble Ca^+2^ causes buffering in cheese which subsides with the solubilization of colloidal calcium phosphate (CCP) during ripening (Hassan, Johnson, & Lucey, 2004). The higher pH levels at the beginning of the storage in our direct acidified cheese samples could be due to buffering effect of CCP. During the storage, with the ongoing proteolysis and solubilization of CCP, it was possible that a decrease in pH occurred due to the loss of buffering. The pH decrease in RC was more remarkable. Rabbit rennet extracts obtained in our laboratory might not be free from lipases as the commercial rennets. Therefore, the lower pH values observed in RC with storage could be due to lipolysis and formation of free fatty acids. Cheese yield of the coagulants is shown in Table 1. The maximum yield was obtained in CC which was associated with its higher moisture content, and moisture‐adjusted yield levels were similar for CC, RC, and CLC with no significant difference (*p* < .05). Even though the difference is not significant, it should be noted that RC and CLC moisture‐adjusted yield values were numerically higher than that of CC. In a study of Cheddar cheese produced by camel and calf chymosin, although yield did not differ significantly, moisture‐adjusted yield for camel chymosin was numerically higher than that of calf chymosin as observed in our study (Bansal et al., 2009). At the beginning of the storage period, CC samples had the highest moisture content in comparison with CLC and RC (Figure 1). Throughout the storage water exudation was observed inside the vacuum packages of cheese samples, and there was a significant decrease (*p* < .05) in moisture content of CC and RC. Maximum water loss occurred in CC samples. Water exudation during storage is a common phenomenon for cheeses with high moisture such as white cheese and feta. High MNFS, low FDM, high protein‐to‐fat ratio, low pH, changes in mineral balance, storage time, and proteolysis are the factors that cause water release from those cheeses. Samal, Pearce, Bennett, and Dunlop (1993) showed that there was a significant increase in the amount of exudate at vacuum‐packed feta cheese by the increase in rennet and storage time and that was correlated with proteolysis. It was suggested that, with the proteolysis of αs_1_‐CN, casein network weakens and gradually disintegrates, releasing moisture held in its interstices as exudate. Kılıç, Kuleasan, Eralp, and Karahan (2009) produced three batches of Turkish white cheese using different starter cultures and measured their total solids content during 120 days of storage. Some fluctuations were observed but according to the trend; a general increase in dry matter content was obvious after 120‐days storage. Irigoyen, Izco, Ibanez, and Torre (2001) studied total protein‐to‐dry matter ratio during 180 day in Roncal cheeses which manufactured with lamb rennet, and cheese samples exhibited a decrease in protein‐to‐dry matter ratio in that storage period. Sample protein contents were measured during 90 days of storage period. According to the results, RC samples contained the highest, and CC samples contained the lowest protein values among these three cheese samples during storage period.

**Table 1:** Composition, yield, and pH of white cheeses made with calf chymosin, camel chymosin, and rabbit rennet

Parameters	Coagulant sources		
	Calf	Rabbit	Camel
Moisture (%)	59.09 ± 0.01^a^	54.09 ± 0.01^b^	53.11 ± 0.03^b^
Fat (%)	18.00 ± 0.01^a^	21.66 ± 0.57^b^	23.00 ± 2.00^c^
Protein (%)	16.82 ± 0.01^a^	19.58 ± 0.01^b^	16.92 ± 0.05^a^
Salt (%)	3.66 ± 0.26^a^	3.58 ± 0.35^a^	3.19 ± 0.13^a^
SM (%)	6.19 ± 0.40^a^	6.61 ± 0.64^a^	6.01 ± 0.21^a^
MNSF (%)	72.06 ± 0.02^a^	69.04 ± 0.02^b^	68.97 ± 0.03^b^
FDM (%)	43.99 ± 0.02^a^	47.17 ± 0.92^b^	49.05 ± 3.00^c^
Yield (kg cheese 100 kg^−1^ milk)	14.08 ± 0.01^b^	12.86 ± 0.01^a^	12.71 ± 0.01^a^
Moisture adjusted yield (kg cheese 100 kg^−1^ milk)^a^	12.28 ± 0.01^a^	12.59 ± 0.01^a^	12.71 ± 0.01^a^
pH			
1 day	5.81 ± 0.01^a^	5.84 ± 0.01^a^	5.86 ± 0.01^a^
15 days	5.74 ± 0.17^a^	5.79 ± 0.01^a^	5.85 ± 0.01^a^
30 days	5.68 ± 0.01^a^	5.51 ± 0.01^a^	5.33 ± 0.01^a^
60 days	5.09 ± 0.01^a^	4.57 ± 0.01^b^	5.23 ± 0.02^a^

SM, salt in moisture; MNFS, moisture in nonfat substance; FDM, fat in dry matter.

Values are means ± standard deviations; means within a row with different superscript letters are significantly different (LSD, *p* < .05).

a Adjusted to 53.11% moisture.

### 3.2 Proteolysis of cheese

**Figure 2:**
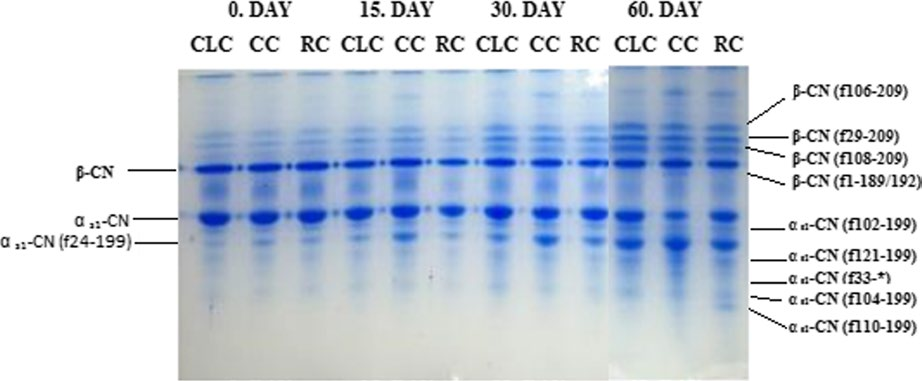
Urea polyacrylamide gel electrophoretograms of CLC, CC, and RC samples after 1, 15, 30, 60 days of storage

Water‐insoluble fractions of cheese samples during storage are displayed in Figure 2. β‐CN was hydrolyzed mainly to β‐CN (f29‐209) (γ1), β‐CN (f106‐209 (γ2), and β‐CN (f108‐209) (γ3). Degradation of β‐CN was detected at all stages of ripening but the intensity of observed bands increased in parallel to the development of ripening due to progress of proteolysis. After the mid‐stage of storage, β‐CN (f1‐189/192) band formation occurs in CC cheese samples. In our study, αs1‐CN(f24–199), αs1‐CN(f102–199), CN(f104–199), CN(f121–199), CN(f110–199) peptides (Figure 2) were identified during ripening period. Similar bands were also reported in the other studies as well (Bansal et al., 2009; Creamer, 1991; Marcos, Esteban, Leon, & Fernandez‐Salguero, 1979; McSweeney, Pochet, Fox, & Healy, 1994; Mooney, Fox, Healy, & Leaver, 1998). In CC cheese samples, the intensity of the band corresponding to αs1‐CN started to decrease after mid‐point of storage; however, intensity of the band representing α_s1_‐CN (f24–199) increased at the same ripening period. The hydrolysis of α_s1_‐CN in RC cheese is lower during 30 days of storage compared to CC and CLC cheese samples which indicates a slower proteolysis in the RC samples. After 30‐days storage, α _s1_‐CN fractions displayed similar degradation patterns in CLC and RC cheese samples but CC cheese samples exhibit higher degradation rates compared to other cheese types.

### 3.3 Rheological and textural properties of white cheese

**Figure 3:**
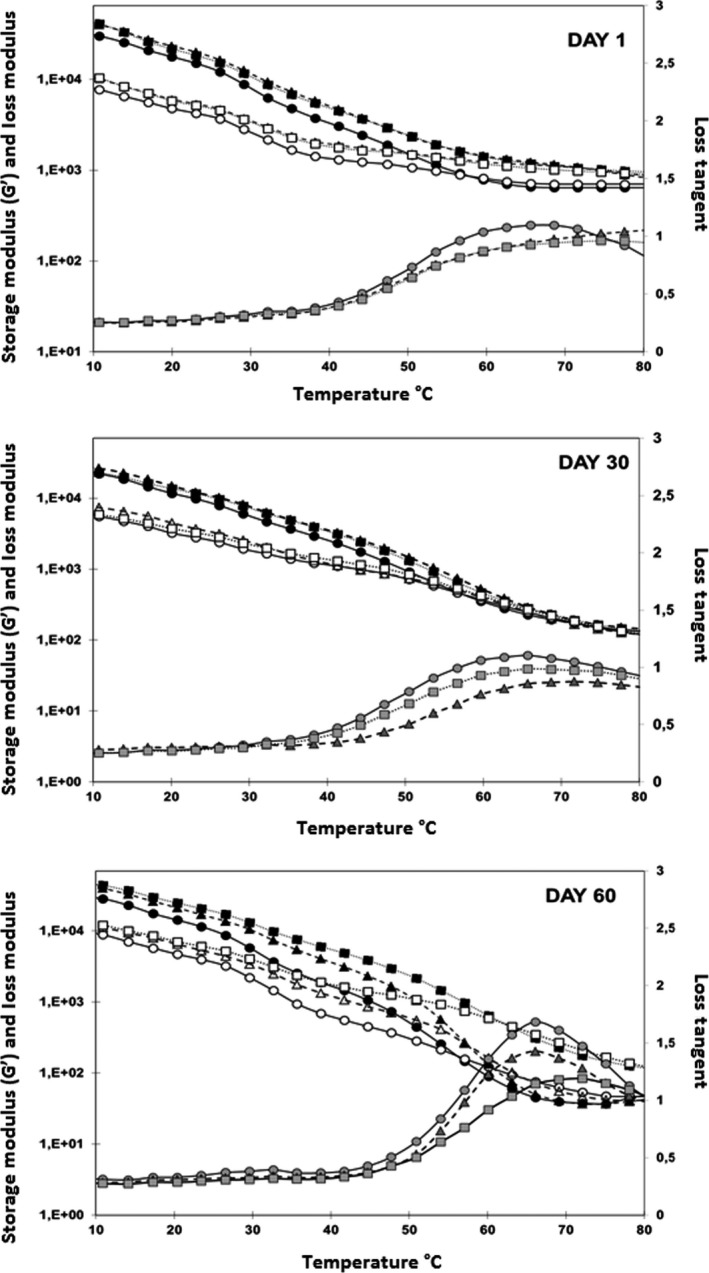
SAOS temperature sweep test results of white cheeses made with calf, rabbit, and camel coagulants during 60 days of storage. Storage modulus (black symbols), loss modulus (open symbols), and loss tangent (gray symbols) of white cheeses made with calf (○), rabbit (∆), and camel (□) coagulants at day 1, day 30, and day 6. Results are the means of replicates

Storage and loss moduli values of RC cheese at day 1 were similar to CLC, while CC exhibited the lowest values (Table 2). Dynamic moduli values indicate the total number and strength of the bonds in cheese matrix (Lucey, Johnson, & Horne, 2003). After 30 days of storage, dynamic moduli values of all cheese samples decreased significantly; however, RC showed the higher storage and loss moduli values as compared to CLC and CC. The decrease in storage and loss modulus shows a reduction in the number of the bonds in protein matrix which could be due to proteolysis. There was an increase in the dynamic moduli values measured at 10°C of all cheese samples at 60th day; CLC being the highest, followed by RC, and CC being the lowest. However, heating reduced both storage and loss moduli to a level lower than day 30 for CC and RC, while CLC had higher moduli than both. CC again had the lowest moduli values at day 60. The increase in moduli was most likely due to higher dry matter of cheese samples at day 60 as a result of water loss during storage. However, cheese samples were more meltable as seen from LT values and reduced moduli at 55°C. Meltability of the cheese has been shown to increase in previous studies due to storage‐related proteolysis (Ak & Gunasekaran, 2002). Temperature at max LT value was higher for RC and CLC than CC at day 1. CC showed higher LT values throughout the storage (Figure 3).

**Table 2:** SAOS temperature sweep test results of white cheeses made with calf, rabbit, and camel coagulants during 60 days of storage

Storage time (days)	Coagulant source	Storage modulus (*G*′) (Pa)		Loss modulus (*G*″) (Pa)		
		10°C	55°C	10°C	55°C	LTmaxT^a^
1	Calf	31,400 ± 254^b,B^	983 ± 80^b,A^	7,980 ± 138^b,C^	915 ± 91^b,A^	68 ± 0^b,A^
	Rabbit	41,800 ± 424^a,B^	1,785 ± 27^a,C^	10,400 ± 113^a,B^	1,380 ± 113^a,A^	84 ± 2^a,A^
	Camel	43,600 ± 410^a,A^	1,795 ± 62^a,A^	10,790 ± 114^a,B^	1,340 ± 24^a,A^	78 ± 6^a,A^
30	Calf	22,800 ± 537^b,C^	550 ± 43^b,B^	5,670 ± 876^b,D^	527 ± 70^a,B^	65 ± 0^a,A^
	Rabbit	26,400 ± 183^a,C^	829 ± 11^a,B^	7,390 ± 593^a,C^	544 ± 71^a,B^	71 ± 0^a,B^
	Camel	23,000 ± 424^b,B^	728 ± 21^a,C^	5,880 ± 183^b,C^	584 ± 12^a,C^	67 ± 4^a,B^
60	Calf	29,550 ± 459^c,B^	210 ± 9^c,C^	9,210 ± 154^b,B^	195 ± 2^c,C^	67 ± 0^b,A^
	Rabbit	40,800 ± 395^b,B^	441 ± 11^b,C^	11,400 ± 989^b,B^	360 ± 6^b,C^	66 ± 0^b,B^
	Camel	43,750 ± 374^a,A^	1,114 ± 12^a,B^	12,050 ± 106^a,AB^	801 ± 56^a,B^	69 ± 0^a,B^

Values are means ± standard deviations.

^a,b,c^Means within a row with different superscript letters are significantly different between coagulant source types (LSD, *p* < .05).

^A,B,C^Means within a column with different superscript letters are significantly different between different storage times (LSD, *p* < .05).

a Temperature at maximum loss tangent.

Texture profile analysis results of cheese samples are given in Table 3. CC had the lowest hardness at day 1, while RC and CLC were similar. CLC exhibited higher hardness values throughout the storage, while CC showed the lowest values. In previous studies using camel chymosin, it was also demonstrated that cheeses made with camel chymosin had higher hardness at storage which was contributed to its low proteolytic activity (Bansal et al., 2009; Govindasamy‐Lucey et al., 2010; Grant, 2011; Kappeler et al., 2006). Our results show that RC was a compatible enzyme with CLC, exhibiting similar hardness and dynamic moduli values at certain storage times. In contrast with the dynamic moduli values, there was an increase in hardness values of all samples after 30 days of storage, with a similar trend as day 1, CC having the lowest hardness. However, cheese samples were very adhesive as seen in Table 3, creating a pasty, stiff texture which could increase hardness levels obtained by a destructive compression test. We could say that dynamic moduli values revealed better the weakening of the protein matrix in cheese at that point. Hardness values decreased at day 60, CLC having the higher hardness than RC, and CC had the lowest value. CC showed lowest gumminess and chewiness values at day 1, while RC and CLC were similar. CLC had the highest gumminess and chewiness values as compared to other cheese types at day 60.

**Table 3:** Texture profile analysis results of white cheeses made with calf, rabbit, and camel coagulants during 60 days of storage

TPA parameters	Coagulant source	Storage time (days)		
		1	30	60
Hardness (g)	Calf	2,423 ± 120^b,C^	5,534 ± 80^b,A^	1,106 ± 71^c,B^
	Rabbit	4,041 ± 385^a,A^	6,320 ± 21^a,B^	2,335 ± 185^b,C^
	Camel	4,842 ± 388^a,A^	6,479 ± 547^a,B^	3,844 ± 159^a,C^
Adhesiveness (g s)	Calf	−54 ± 4.3^a,A^	−239 ± 12.4^a,B^	−19 ± 7.2^a,A^
	Rabbit	−75 ± 0.56^a,A^	−320 ± 70^b,B^	−19 ± 9.8^a,A^
	Camel	−77 ± 2.1^a,A^	−214 ± 120^a,B^	−22 ± 4.1^a,A^
Gumminess	Calf	540 ± 30^b,B^	1,315 ± 67^a,A^	526 ± 0^b,B^
	Rabbit	981 ± 52^a,A^	1,018 ± 44^b,A^	814 ± 93^b,A^
	Camel	1,073 ± 40^a,BC^	1,328 ± 426^a,B^	1,652 ± 58^a,A^
Chewiness	Calf	280 ± 20^b,B^	833 ± 10^a,A^	334 ± 6^b,B^
	Rabbit	493 ± 60^a,A^	406 ± 31^b,A^	455 ± 87^b,A^
	Camel	543 ± 46^a,C^	1,037 ± 299^a,B^	1,288 ± 67^a,A^

Values are means ± standard deviations.

^a,b,c^Means within a row with different superscript letters are significantly different (LSD, *p* < .05).

^A,B,C^Means within a column with different superscript letters are significantly different (LSD, *p* < .05).

### 3.4 Sensorial analysis

Sensory evaluation is important for determining the eating quality of cheese and its consumer acceptability (Delahunty & Drake, 2004). From past to present, many of coagulant sources were tried out in cheese‐making but only some of them were accepted due to sensorial characteristics of produced cheeses. The sensory analyses results of CC, RC, and CLC samples are given in Table 4. At the beginning of the storage; taste, appearance, and odor of all three cheese samples were similar, while structure of CC was found inferior to RC and CLC. There were no significant differences between the appearance of RC and CLC (*p* > .05) during 60‐days storage. At 30 days of storage, there was a significant decrease in all sensory analysis results of CC. Odor and taste scores of samples were gradually decreased in general during storage, RC having the highest scores after 60 days. Commercial calf chymosin is the most popular coagulant source in cheese‐making but our physicochemical and sensory analysis show that RC cheese samples exhibit better physicochemical and sensorial characteristic than the samples made with calf chymosin and similar quality profile to that of camel samples.

**Table 4:** Sensory analysis results of white cheeses made with calf chymosin, camel chymosin, and rabbit rennet after 1, 15, 30, 60 days of storage

Storage time (days)	Coagulant source	Parameters				
		Outer appearance	Inner appearance	Structure	Odor	Taste
1	Calf	4.25 ± 0.88^b,AB^	4.50 ± 0.53^a,AB^	3.87 ± 0.83^b,A^	4.87 ± 0.35^a,AB^	4.62 ± 0.51^a,A^
	Rabbit	4.87 ± 0.35^ab,A^	4.75 ± 0.46^a,B^	4.25 ± 0.70^ab,AB^	5.00 ± 0.00^a,A^	4.87 ± 0.33^a,A^
	Camel	5.00 ± 0.00^a,A^	4.87 ± 0.35^a,A^	4.62 ± 0.51^a,A^	5.00 ± 0.00^a,A^	4.87 ± 0.35^a,A^
15	Calf	4.62 ± 0.74^a,A^	4.75 ± 0.70^a,AB^	4.37 ± 0.74^ab,A^	5.00 ± 0.00^a,A^	4.56 ± 0.49^a,AB^
	Rabbit	4.62 ± 0.51^a,AB^	4.50 ± 0.53^a,AB^	3.87 ± 0.83^b,A^	5.00 ± 0.00^a,A^	4.75 ± 0.46^a,A^
	Camel	4.75 ± 0.70^a,AB^	4.87 ± 0.35^a,A^	4.87 ± 0.35^a,A^	5.00 ± 0.00^a,A^	4.71 ± 0.45^a,AB^
30	Calf	3.75 ± 0.70^b,B^	4.50 ± 0.75^a,AB^	3.87 ± 0.99^b,A^	4.62 ± 0.51^a,BC^	4.00 ± 1.06^a,BC^
	Rabbit	4.50 ± 0.53^a,AB^	4.87 ± 0.35^a,A^	4.50 ± 0.53^a,B^	4.75 ± 0.70^a,A^	4.50 ± 0.75^a,A^
	Camel	4.50 ± 0.75^a,ABC^	4.87 ± 0.35^a,A^	4.75 ± 0.46^a,A^	4.75 ± 0.46^a,AB^	4.12 ± 0.83^a,B^
60	Calf	4.00 ± 0.75^a,AB^	4.12 ± 0.64^b,CD^	4.25 ± 0.70^b,A^	4.50 ± 0.53^a,CD^	4.12 ± 0.64^a,AB^
	Rabbit	4.25 ± 1.03^a,AB^	4.50 ± 0.75^ab,AB^	4.25 ± 0.70^b,AB^	5.00 ± 0.00^ab,A^	4.50 ± 0.92^a,A^
	Camel	4.25 ± 0.70^a,BC^	4.75 ± 0.46^a,A^	5.00 ± 0.00^a,A^	4.75 ± 0.46^a,AB^	4.50 ± 0.75^a,AB^

Values are means ± standard deviations.

^a,b^Means within a row with different superscript letters are significantly different (LSD, *p* < .05).

^A,B,C,D^Means within a column with different superscript letters are significantly different (LSD, *p* < .05).

## 4 CONCLUSIONS

Three batches of white cheeses were made using camel chymosin, calf chymosin, and rabbit rennet extract using the same processing conditions without any starter culture addition. RC and CLC exhibited higher hardness and dynamic moduli values throughout the storage as compared to CC. Moisture loss due to water release was observed in cheese samples during 60‐days storage. Although moisture levels of cheese samples were similar by day 60, CC had much lower hardness and dynamic moduli values than CLC and RC. Softening of the cheese texture during storage is associated with proteolysis, so the higher hardness of RC and CLC could be an indicator of low proteolytic activity of rabbit and camel chymosins. While the overall sensory appearance and structure were better for CLC, the highest odor and taste scores were obtained by RC during 60 days of storage. Commercial chymosins have several superior properties such as better clotting and low proteolytic activity over rennets because of its pepsin and lipase content in addition to chymosin. In our study, we used rabbit rennet as a coagulant source besides commercial chymosins, and the results revealed that RC has comparable properties to CLC having similar textural and rheological results. Therefore, rabbit rennet might be considered as a valid alternative to the milk coagulant enzymes presently available in the market.
